# Modeling linear and nonlinear viscoelastic oscillatory rheometric stress-strain hysteresis of asphalt binders

**DOI:** 10.1038/s41598-024-78551-8

**Published:** 2024-11-18

**Authors:** Aboelkasim Diab, Lingyun You, Ameen Topa, Nikhil Saboo, Mayank Sukhija, Ahmed Awed

**Affiliations:** 1 Department of Civil Engineering, Aswan University, Aswan, 81542 Egypt; 2 School of Civil and Hydraulic Engineering, Huazhong Univ. of Science and Technology, Wuhan, 430074 Hubei China; 3 Department of Maritime Technology, Faculty of Ocean Engineering Technology and Informatics, Universiti Malaysia Terengganu, Kuala Terengganu, 21030 Malaysia; 4 Department of Civil Engineering, Indian Institute of Technology Roorkee, Roorkee, Uttarakhand 247667 India; 5 School of Civil and Construction Engineering, Oregon State University, Corvallis, OR 97331 USA; 6 Public Works Engineering Department, Faculty of Engineering, Mansoura University, Mansoura, 35516 Egypt

**Keywords:** Asphalt binders, Linear/nonlinear viscoelastic behavior, Hysteresis stress-strain loops, FEC hyperelastic model, Numerical FE modeling, Engineering, Civil engineering, Mechanical engineering

## Abstract

Understanding the complex stress-strain hysteresis behavior of asphalt binders under varied conditions is critical for optimizing pavement performance. This study addresses the challenge by analyzing and modeling asphalt binder responses in oscillating shear mode across different aging states (unaged, short-term aged, and long-term aged), stretch amplitudes, frequencies, and temperatures. Fifty-three stress-strain hysteresis loops were meticulously analyzed, revealing distinct stress paths relative to applied stretch levels. A nine-parameter parallel rheological framework model was developed, integrating a four-parameter eight-chain (FEC) hyperelastic model in one network and a FEC hyperelastic model with a linear viscoelastic flow element in series in another. This constitutive model was implemented in LS-DYNA finite element simulations to predict experimentally-measured stress-strain hysteresis loops accurately. The research demonstrates the model’s capability to simulate both linear and nonlinear viscoelastic responses of asphalt binders across a wide range of environmental and loading conditions. This approach significantly enhances our ability to capture and understand the stress-strain behavior critical for asphalt pavement durability and performance optimization.

**Supplementary Information:**

The online version contains supplementary material available at 10.1038/s41598-024-78551-8.

## Introduction

The asphalt mixture exhibits a composite structure consisting of various materials, with its behavior being highly dependent on time and temperature. The performance of the asphalt mixture system is ultimately influenced by the behavior of its individual constituents^1–5^. The interaction between temperature and excitation inputs (strain rate and strain amplitude) introduces complexity in describing the mechanical behavior of asphalt materials^6^. Temperature, stretch (or strain) range, and stretch rate (or frequency) significantly alter the mechanical properties of these viscoelastic materials, affecting their internal structure and nonlinearity, thus contributing to the complexity of their behavior. Understanding the mechanical properties of asphalt materials under various loading conditions is fundamental^7^. Therefore, developing a modeling approach that accurately reflects both linear and nonlinear viscoelastic behavior remains a challenging task^8,9^. Researchers continually strive to develop new testing methods and modeling approaches to better understand such behavior.

Asphalt pavements are designed to withstand a wide range of traffic loads and climatic conditions. Under external loading, the asphalt layers can exhibit viscoelastic-plastic behavior, depending on the induced stretch level^10–12^. Inasmuch at infinitesimal strain ranges, the macro-scale asphalt mixture remains in the linear viscoelastic region. However, the thin film of asphalt binder between aggregate particles may experience larger strains, extending into the nonlinear viscoelastic or plastic domains^10^. The mechanical behavior of the asphalt mixture is primarily dictated by its constituents, particularly the asphalt binder, which governs the response through its rheological properties. Therefore, understanding the mechanical behavior of the asphalt binder is crucial for comprehending the overall mechanical characteristics of asphalt mixtures^13,14^.

Measuring and modeling the mechanical behavior is essential for assessing the rheological behavior of asphalt materials and understanding their sensitivity to various influencing factors^15,16^. While testing asphalt binder behavior in the linear viscoelastic region is more convenient, interpreting the nonlinear response from laboratory tests remains challenging^6,17,18^. Oscillatory shear tests, which are commonly performed using stress/strain-controlled rheometers, are used to grade asphalt binders and evaluate their viscoelastic rheological properties. This rheometric shear setup is a key component of the Superpave binder grading system^6,19^. Asphalt materials display complex stress-strain patterns, making them troublesome to be captured and modeled. The inherent viscoelastic-plastic mechanical response, characterized by dependencies on strain rate, strain level, and temperature, complicates the modeling of time-dependent stress-strain behavior^20–22^.

Numerous literature-based attempts have proposed modeling approaches to accurately represent the rheological behavior of asphalt materials, often focusing on linear viscoelastic behavior^23–25^. Historically-developed models have included both linear and nonlinear viscoelastic characteristics, but their success depends on specific loading conditions^8,10–12,15,24–35^. Most literature-based approaches have successfully captured the full response within certain limits and loading environments^32,36,37^. Stress-strain behavior, which dictates material deformation characteristics in its intended application, is crucial for supporting modeling relationships that govern the behavior and pattern^36^.

Stress-strain behavior in both linear and nonlinear viscoelastic domains can be assessed using dynamic mechanical analysis techniques^38^. The complex hysteresis behavior of stress-strain loops in both domains has posed significant challenges to modeling attempts. For example, Diab et al.^20,21^ promoted modeling dynamic shear rheological outcomes across various conditions, and they proposed to expand the testing conditions for the rheometric hysteresis of asphalt binders to validate the models under a broader range of conditions. Padmarekha et al.^24^ applied frame-invariant nonlinear constitutive modeling under limited conditions to depict the waveform response of tested binders. However, the asphalt binder’s response was only predicted under limited conditions, and there is no clear conclusion that the model can predict hysteresis behavior across a broad range of experimental data. Di Benedetto^28^ developed an enhanced nonlinear viscoelastic model based on the 2S2P1D linear viscoelastic framework. The study found that the proposed model effectively characterizes the nonlinear behavior of asphalt concrete (AC) mixtures subjected to small strain states. Masad et al.^11^ developed a method to analyze the nonlinear viscoelastic behavior of original and aged asphalt binders tested under varying stresses and temperatures using a dynamic shear rheometer (DSR). Their numerical modeling closely matched the experimental results. However, the study did not utilize the hysteresis behavior from rheometric measurements of asphalt binders, which accounts for both linear and nonlinear behavior. Hajikarimi et al.^14^ proposed a generalized fractional nonlinear viscoelastic model to predict the creep and recovery behavior of base and modified asphalt binders, yielding reasonable predictions under specific testing conditions. Sun et al.^30^ utilized the full Schapery nonlinear viscoelastic (NLVE) model along with LVE properties from frequency sweep tests to develop a framework for accurately extracting the irrecoverable and recoverable responses of binders from the multiple stress creep recovery (MSCR) test. Their study demonstrated that this framework effectively characterizes the NLVE and irrecoverable behaviors of both base and polymer-modified binders. Sadeq et al.^29^ applied a nonlinear plasto-viscoelastic (NPVE) approach to distinguish between recoverable and irrecoverable strains in the MSCR test for warm mixed asphalt binders. Their analysis showed that the NPVE approach captures a greater percentage of recovery than the NLVE method. Delgadillo et al.^27^ established a nonlinear constitutive relationship for asphalt binders based on creep and recovery tests at different stress levels and loading durations. Their findings demonstrated that a nonlinear power law function effectively models the binders’ creep response.

Although existing work acknowledges prior studies on linear and nonlinear viscoelastic modeling of asphalt materials, it is evident that these models do not effectively predict the oscillatory rheometric stress-strain hysteresis behavior of asphalt materials, pertaining both linear and nonlinear responses. Additionally, the intricate characteristics of asphaltic materials and their diverse responses to external stimuli reveal significant research gaps in asphalt binder rheology, which impede a thorough understanding of material behavior under varying conditions^32^. In rheometric tests of asphalt binders, the small-amplitude oscillatory shear mode is employed in the linear viscoelastic region, while large-amplitude oscillatory shear mode is utilized in the nonlinear viscoelastic region. The increase in applied strain amplitude at a specific frequency and temperature indicates a discernible transition between these two regimes. However, current rheometric stress-strain hysteresis testing protocols for large-amplitude oscillatory shear and associated rheological indices do not yield a conclusive rheological evaluation of asphalt binders^13^. There is a lack of knowledge regarding the effects of temperature, strain range, and strain rate on the mechanical properties and internal structure of asphalt binders, particularly under nonlinear viscoelastic conditions derived from rheometric stress-strain hysteresis of asphalt binders. Existing methods predominantly address linear viscoelastic behavior, leaving a substantial gap in accurately capturing the linear and nonlinear viscoelastic oscillatory rheometric stress-strain hysteresis behaviors of asphalt materials. Oscillatory shear tests, although commonly employed, pose challenges in interpreting nonlinear responses, as existing models largely overlooked the complex stress-strain patterns and hysteresis behavior observed under oscillatory loading conditions. These gaps highlight the necessity for advanced modeling approaches that can encompass the full spectrum of oscillatory rheometric stress-strain hysteresis responses of asphalt binders under a varied range of practical conditions.

## Theoretical background

**Fig. 1:**
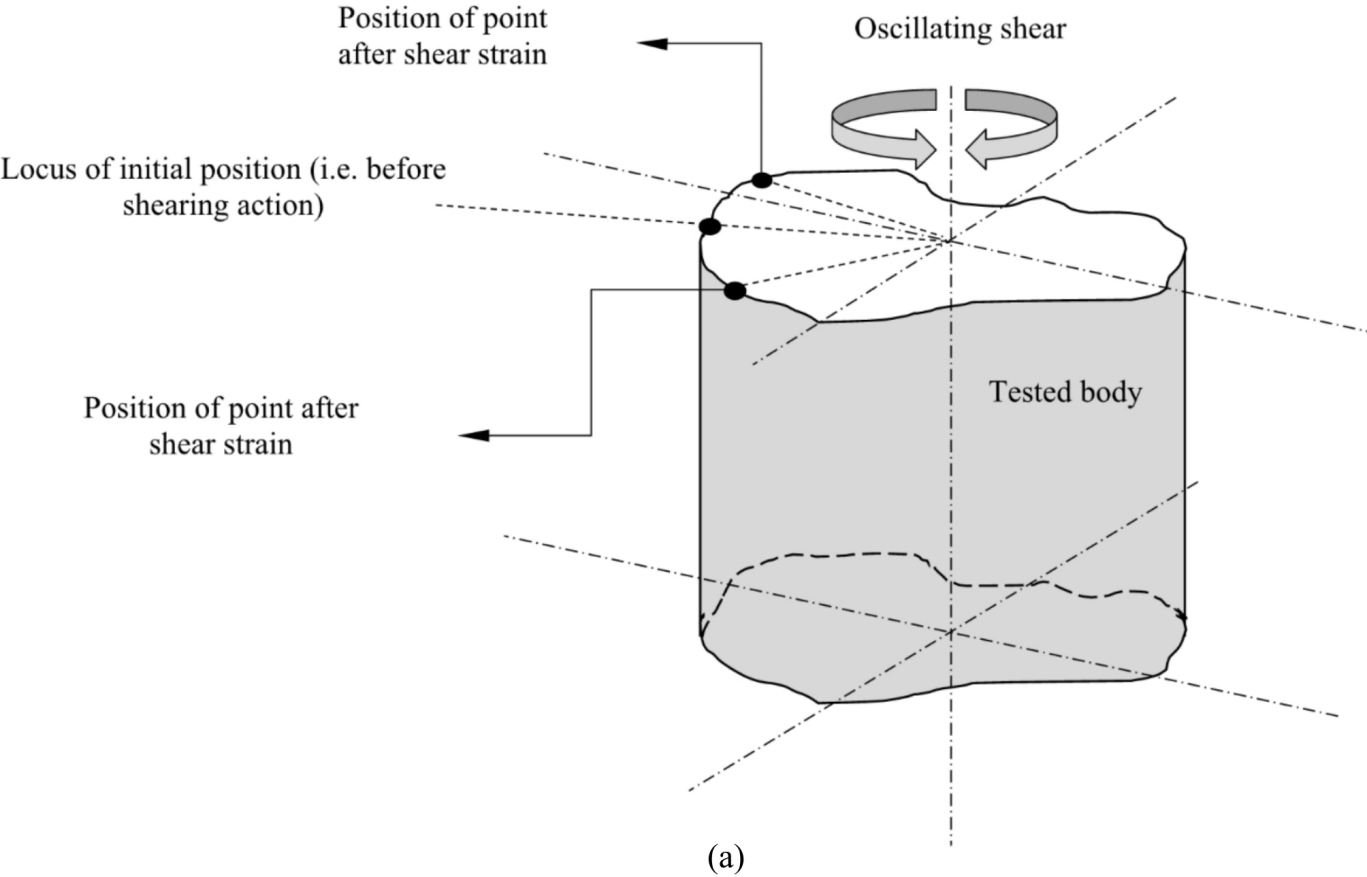

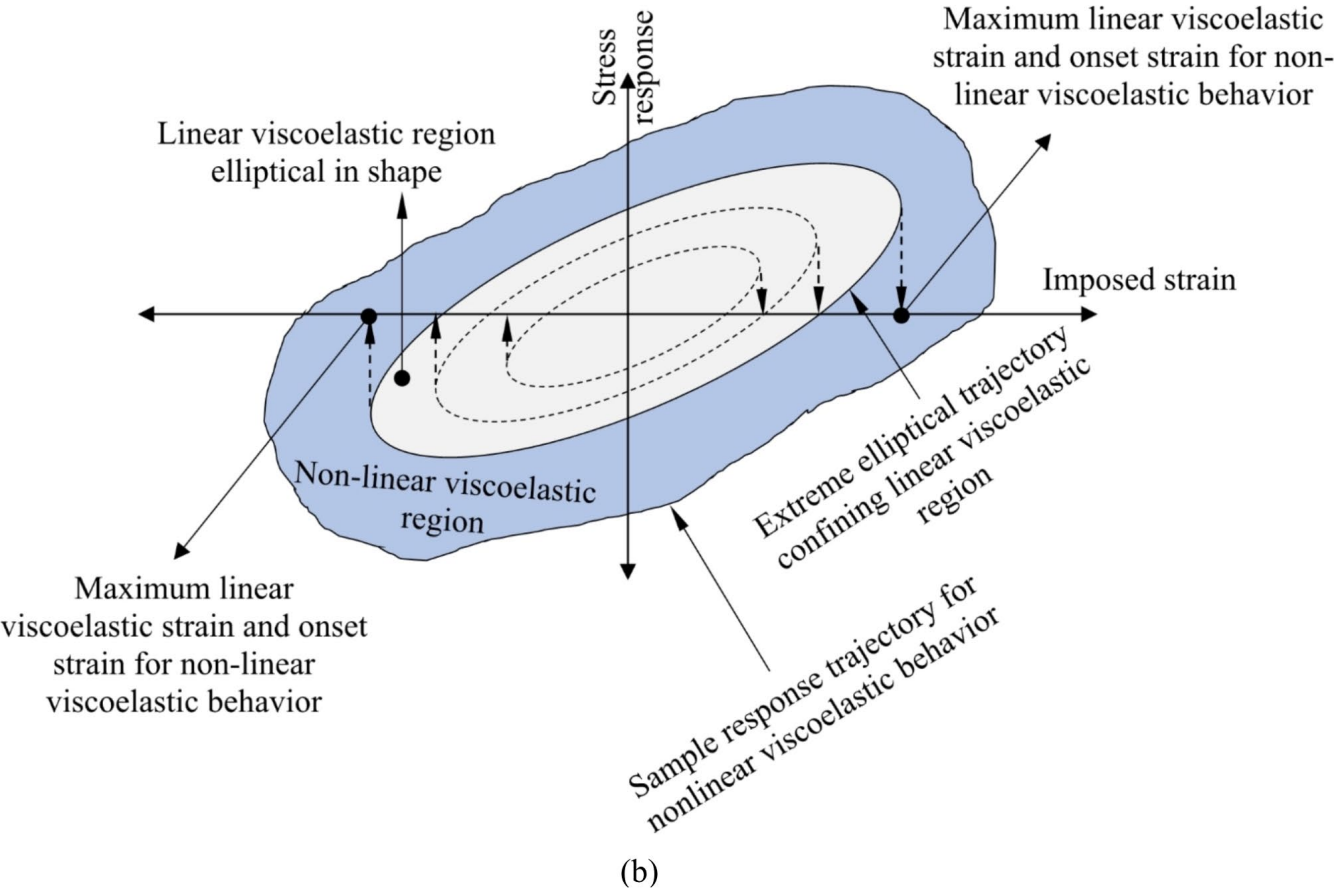
Rheometric stress-strain behavior of asphalt binder (**a**) asphalt specimen under oscillating shear mode, and (**b**) analogous stress–strain hysteresis orbit for linear viscoelastic and nonlinear viscoelastic responses.

Linear viscoelastic properties of asphalt binders can be identified using dynamic shear analysis to determine the strain limits within which asphalt samples are tested. Dynamic mechanical methodologies can also extend to provide an approach for nonlinear characterization of asphalt materials^39^. Initially, as asphalt material undergoes strain within the linear region, it exhibits linear viscoelastic behavior; with greater perturbations, it transitions to nonlinear viscoelastic behavior^40,41^. Figure 1 illustrates general features of linear and nonlinear responses of the asphalt material undergoing small- and large-amplitude oscillatory shear loading. Obtaining the stress-strain hysteresis loop involves twisting binder or mixture specimens in oscillatory shear mode under specific conditions, including temperature and excitation inputs (e.g., strain amplitudes and rates).

The linear and nonlinear viscoelasticity of materials can be graphically illustrated by the eminent Lissajous plot (stress vs. strain plot)^42^. Distinguishing between linear and nonlinear viscoelastic behaviors of asphalt binder through the demarcation between the two regions binder is not a trivial task^6,11,13,43^. The nonlinear regime is reached when excitation exceeds the permissible strain range of the linear viscoelastic region, leading to increased non-linearity as deformation intensifies^44^. Damage in the asphalt material manifests as a significant reduction in stiffness and degradation of the material’s mechanical response due to irreversible microstructural changes during the consecutive cycles of loading^35^. Under linearly viscoelastic behavior in strain-controlled mode, the conjugated stress waveform follows a sinusoidal pattern led by a phase angle (*δ*) from the sinusoidal input strain. In nonlinear conditions, the stress response to sinusoidal input strain is no longer sinusoidal, and the stress–strain pattern is not perfectly elliptic, complicating the characterization of different viscoelastic materials^44^.

Broadly. viscoelastic properties of materials, such as asphalt binder, are depicted in terms of responses to sinusoidal inputs (cyclic strain/stress). From binder testing in the classical linearized theory of viscoelasticity, $\:{G}^{*}$ (complex modulus) and $\:\delta\:$ (phase angle) as functions of *ω* (angular frequency) and T (temperature) are most predominant indices determined from sinusoidal shear oscillations to evaluate asphalt material performance^19^. In the linear viscoelastic regime, the binder’s modulus is independent of oscillation amplitude; however, it changes for larger amplitudes or perturbations that overshoot this region^42^.

For strain-controlled tests, let the applied strain be a harmonic function of time with an amplitude of $\:{\varepsilon\:}_{0}$. While ω is the angular frequency of oscillation, the strain function $\:\varepsilon\:\left(t\right)$ is given as follows^19^:

1 $$\:\varepsilon\:\left(t\right)={\varepsilon\:}_{0}{e}^{i\omega\:t}$$

For linear viscoelastic measurements, a band-limited strain signal is used as input based on AASHTO T 315, and the stress response $\:\sigma\:\left(t\right)$ is monitored. The stress response waveform $\:\sigma\:\left(t\right)$ is a function of the driving strain as follows:

2 $$\:\sigma\:\left(t\right)={\sigma\:}_{0}{e}^{i(\omega\:t+\delta\:)}$$

where $\:{\sigma\:}_{0}$ is the response (stress) amplitude.

Therefore, for a linear viscoelastic material based on the preceding equations, the stress-strain relationship is elliptical, as exhibited in Eq. (3)^45^.

3 $$\:{\left(\frac{\sigma\:}{{\sigma\:}_{0}}\right)}^{2}+{\left(\frac{\varepsilon\:}{{\varepsilon\:}_{0}}\right)}^{2}=2\left(\frac{\sigma\:}{{\sigma\:}_{0}}\right)\left(\frac{\varepsilon\:}{{\varepsilon\:}_{0}}\right)\text{cos}\delta\:+{\left(\text{sin}\delta\:\right)}^{2}$$

The elliptical shape of the stress-strain loop is a consequence of linear viscoelastic behavior, provided the strain is within the linear viscoelasticity threshold. If tested at excitation levels beyond this threshold, the stress-strain orbit in dynamic loading mode will deviate from ellipticity, indicating material nonlinearity^41^. In the dynamic shear rheometer (DSR) test, stress-strain hysteresis loops of the asphalt binder vary in shape depending on testing conditions. Modeling and interpreting the stress-strain behavior from these measurements remain active research areas because the stress-strain loop lacks a specific pattern under non-linear viscoelastic conditions^15^.

A mutual issue in modeling asphalt material behavior is using techniques designed for large condition domains. There is limited knowledge about asphalt binder nonlinearity when different parameters (e.g., stretch level, stretch rate, temperature) contribute to its response. The main concern of this article was the analysis and modeling of the intricate stress-strain hysteresis behavior of asphalt binders in shear mode within the framework of linear and nonlinear viscoelasticity. The motivation underlying this work was to improve modeling and characterization techniques for asphalt materials’ mechanical behavior. The parallel rheological framework (PRF) model was structured to describe the hysteresis stress-strain behavior of asphalt binder in shear testing. Using LS-DYNA, finite element (FE) models were developed by importing user-defined constitutive material model parameters to numerically verify material behavior and model application efficiency in the FE platform. Existing material models for asphalt binder in FE software packages are not always effective; thus, the proposed study integrated constitutive model parameters in the LS-DYNA solver to reliably model rheometric conditions.

## Research gap, main objectives, and novel contributions

This study addresses the need for advanced mechanical modeling of the rheological behavior of asphalt binders. Existing models often fall short in accurately capturing the stress-strain hysteresis behavior of asphalt binders under various oscillating shear conditions, especially across different temperatures, strain amplitudes, and frequencies. This gap in knowledge motivated the authors to develop a comprehensive modeling framework that can accurately represent both linear and nonlinear viscoelastic behavior through achieving the main objectives of this research as follows:

- Implementing small-amplitude and large-amplitude oscillatory testing protocols to record shear stress-strain loops and behavior patterns of asphalt binders across a range of conditions, including varying temperatures and excitation parameters.
- Developing a structure for parallel rheological framework constitutive mechanical model to accurately depict the hysteresis loops of shear stress-strain, thereby enabling comprehensive material characterization in both linear and nonlinear viscoelastic domains.
- Developing a numerical approach to model the rheometric conditions of asphalt binders using a user-defined material model derived from the constitutive framework and implementing this in the LS-DYNA FE environment for simulation purposes.

This study introduces several novel contributions to the field of rheological behavior modeling of asphalt binders:

- Establishing comprehensive small- and large-amplitude oscillatory testing protocols to record shear stress-strain loops across a broad range of conditions, including different temperatures and excitation parameters. This comprehensive approach ensures a thorough understanding of the material’s behavior under various scenarios.
- A parallel rheological framework constitutive model was developed to capture both the linear and nonlinear viscoelastic behavior of asphalt binders. This model uniquely combines FEC hyperelastic and linear viscoelastic flow models to accurately depict the stress-strain hysteresis loops, providing a more robust and detailed characterization of the material.
- Developing a novel numerical approach to simulate the rheometric conditions of asphalt binders using a user-defined material model in the LS-DYNA FE environment. This integration allows for precise simulation and validation of the material’s behavior under dynamic shear loading, demonstrating the practical application of the constitutive model in finite element analysis.
- Validating the proposed model by extensively testing unaged, short-term aged, and long-term aged binder samples under various temperatures and strain rates. This thorough validation process ensures the reliability and applicability of the proposed modeling framework across different aging conditions and testing scenarios.

## Parallel rheological framework constitutive mechanical model

**Fig. 2:**
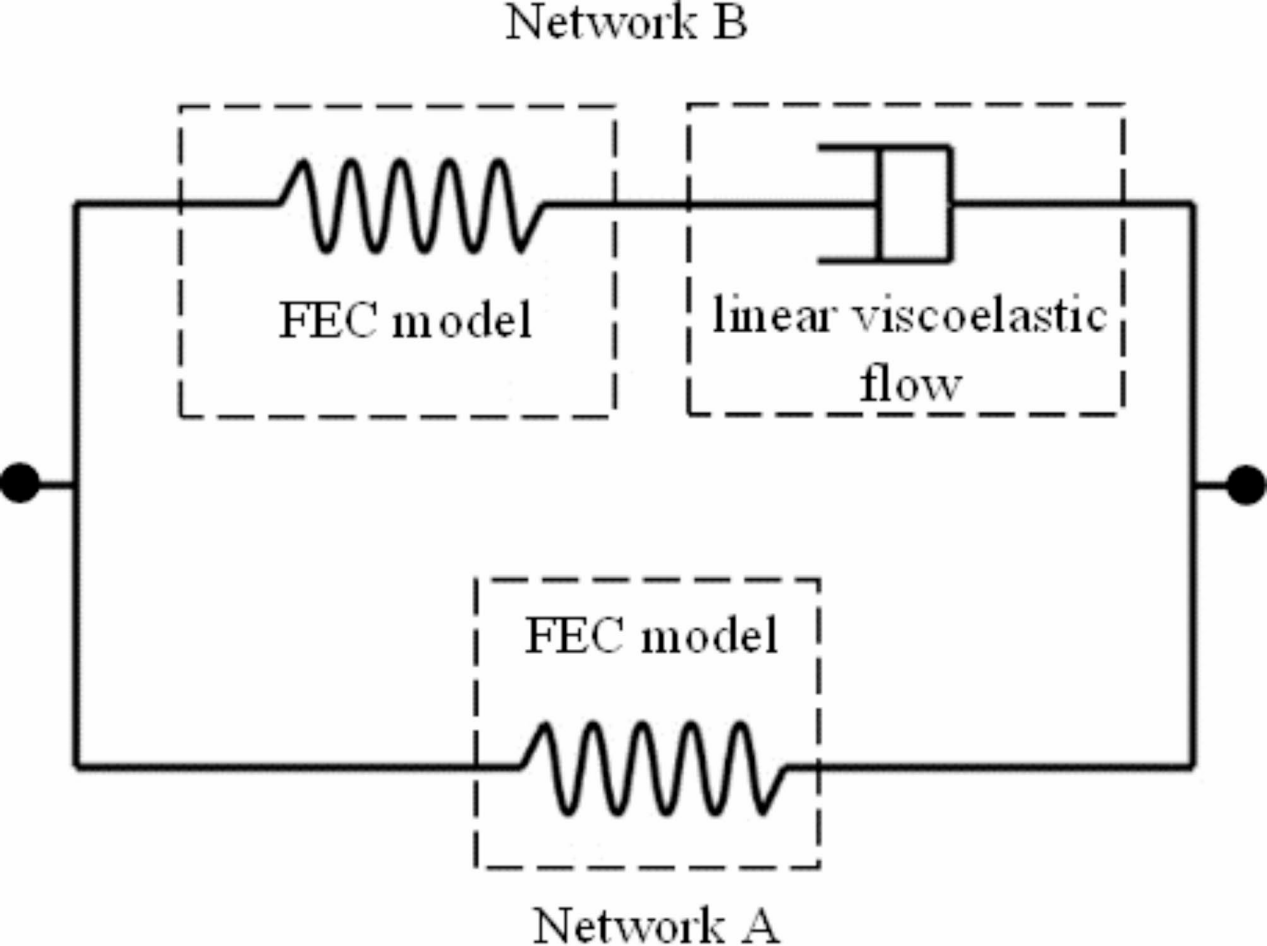
Mechanical structure of the constitutive model.

The comprehensive modeling of linear and nonlinear viscoelastic stress-strain hysteresis behaviors in asphalt binders was achieved through developing a nine-parameter parallel rheological framework model. The structured constitutive mechanical model consists of a parallel combination of two networks, A and B, as depicted in Fig. 2. This model is built upon the arrangement of four-parameter eight-chain (FEC) hyperelastic and linear viscoelastic flow models. Network A basically captures the equilibrium response of the material using a FEC hyperelastic model. Network B is represented by a FEC hyperelastic model in series with a time-dependent element that exhibits linear viscoelastic flow characteristics.

The Cauchy stress $\:\sigma\:$ for the FEC hyperelastic model is formulated as follows^46^:

4 $$\:\sigma\:=\frac{1}{J}\left[{\mu\:}_{1}+\frac{{\mu\:}_{2}}{\stackrel{-}{{\lambda\:}^{*}}}\:\frac{{\mathcal{L}}^{-1}\left(\frac{\stackrel{-}{{\lambda\:}^{*}}}{{\lambda\:}_{L}}\right)}{{\mathcal{L}}^{-1}\left(\frac{1}{{\lambda\:}_{L}}\right)}\right]dev\left[{b}^{*}\right]+k\left(J-1\right)I$$

where, $\:J$ is the determinant of the deformation gradient (= det[F]), $\:{\mu\:}_{1}$ and $\:{\mu\:}_{2}$ are the shear moduli 1 and 2; respectively, $\:{\lambda\:}_{L}$ is the limiting chain stretch, $\:k$ is the bulk modulus of the material,$\:\:{b}^{*}$ is the distortional left Cauchy-Green tensor (=$\:\:{J}^{-2/3}\text{F}{\text{F}}^{\text{T}}$), $\:\stackrel{-}{{\lambda\:}^{*}}$is the applied chain stretch ($\:=\sqrt{\frac{tr\left[{b}^{*}\right]}{3}}$), $\:{\mathcal{L}}^{-1}\left(x\right)$ is the inverse Langevin function (=$\:\:\text{c}\text{o}\text{t}\text{h}\left(\text{x}\right)-\frac{1}{x}$), and *I* is the strain invariant.

For the dashpot, the flow rate $\:{\dot{\gamma\:}}^{p}\:$is given by^47^:

5 $$\:{\dot{\gamma\:}}^{p}=\left(\frac{\tau\:}{{f}_{p}{f}_{{\epsilon}p}{f}_{\theta}\widehat{\tau\:}}\right)$$

where, $\:\tau\:$ is the applied shear stress, $\:\widehat{\tau\:}$ is the shear flow resistance, and $\:{f}_{p},\:{f}_{{\epsilon}p},\:{and\:f}_{\theta}$ are model factors.

The sampling data points of strain-stress loops from dynamic rheometric tests were used to parameterize the material model. The model parameters for various conditions were determined using a nonlinear regression technique to minimize the error between the experimental and modeled stress-strain loops. The coefficient of determination (*R*^*2*^) provided numerical values to appraise the degree of convergence of the model prediction to the experimental data. Further details about the optimization of model parameters are explained in Sect. 6.

## Materials and measured data processing

To investigate the soundness of the constitutive modeling using the parallel rheological framework, multiple experimental stress-strain hysteresis results were collected for unaged, short-term aged, and long-term aged binder samples using the DSR. An experimental database was constructed to characterize the mechanical hysteresis behavior of the tested samples, from which possible characteristics were compiled for model validation. The physical properties of the asphalt binder tested for shear stress-strain hysteresis behavior are listed in Table 1.

The rheometric tests were performed at the asphalt laboratories of the Indian Institute of Technology, Roorkee, India. The samples tested in this study originated from one source of base binder graded as VG30. The unmodified binder underwent short- and long-term aging processes using the rolling thin film oven (RTFO) and pressure aging vessel (PAV) conditions, according to ASTM D 2872^48^ and ASTM D 6521^49^, respectively.

**Table 1:** Physical properties of original base asphalt binder VG30.

Properties	Obtained Results	Specified Limits
Penetration value at 25 °C, 0.1 mm	49	Minimum 45
Viscosity at 60 °C, Poise	3400	2400–3600
Kinematic viscosity at 135 °C, cSt	372	Minimum 350
Flash point, °C	240	Minimum 220
Solubility in trichloroethylene, %	99.8	99
Softening point, °C	53	Minimum 47

An intriguing approach was employed to better characterize the stress-strain patterns and hysteresis behavior of asphalt binders, involving shear excitation measurements. The oscillating hysteresis characterization of asphalt binder samples was determined using the DSR with the parallel plate measurement system. Oscillatory shear tests were performed under various conditions for three replicates per each condition. Based on AASHTO T 315^50^, the stretching level was used as a definite input to diagnose whether the sample would undergo linear or nonlinear behavior. Therefore, a matrix of testing conditions was suggested to capture stress-strain behaviors for model validation.

Oscillatory shear tests are executed within the linear viscoelastic region utilizing small-amplitude oscillatory shear mode or within the nonlinear viscoelastic region employing large-amplitude oscillatory shear mode. A noticeable transition between the linear and nonlinear regimes is observed with an increase in the applied strain amplitude at a designated frequency and temperature. Small amplitude oscillatory shear tests represent the predominant rheological testing methodologies for investigating the linear viscoelastic properties of asphalt binders. For linear viscoelastic measurements, AASHTO T 315 specifies permissible stretch values: 9–15% for the base binder, 8–12% for the short-term aged residue, and 0.8–1.2% for the long-term aged residue. Although various techniques exist for measuring nonlinear viscoelastic properties, an established standard testing procedure for asphalt binders has not yet been developed by researchers. For nonlinear behavior, experimentalists may select higher strain levels than those previously mentioned, based on their discretion. Despite the widespread utilization of large amplitude oscillatory shear rheology across diverse materials, it currently lacks a clear physical interpretation that correlates with the macroscopic rheological response. To date, the protocols for large-amplitude oscillatory shear testing and the corresponding material metrics do not yield a definitive rheological fingerprint for asphalt binders; thus, no established standard for nonlinear rheometric stress-strain hysteresis measurements, as the behavior is beyond the linear viscoelastic region. A wide range of stress-strain conditions was targeted for designing and validating the constitutive modeling approach. This was achieved by testing the material source in unaged, short-term aged, and long-term aged conditions at low and high temperatures, with small and high stretch rates, and imposing stretch levels from very low to very high values. Table 2 outlines the testing conditions conducted on the samples to identify individual stress-strain hysteresis behaviors based on the experimental data. Testing temperature, stretch values, and strain frequencies were integrated as presented in Table 2 to consider their influence on the material’s behavior. Shear oscillation was performed at 1, 10, and 25 Hz. It should be noted that maintaining adherence between the upper/lower plate of the rheometer and sample surfaces at very high stretch rates and/or high stretch values was impractical at both low and high temperatures, so such results were not included or analyzed in this manuscript. Although the data have not been systematically reviewed for potential compliance-related issues, observations of plate slip and/or sample fracture were indicated by a significant decline in torque readings during testing. When plate slip was identified by the operator, the corresponding measurement was excluded from the analysis. In the results presented, it should be noted that the test results for RTFO- and PAV-aged samples at high frequency were omitted from the analysis and are not included in the main body of the paper, as accurate measurement of the stress-strain hysteresis loops could not be conducted due to instability or loss of adhesion between the steel plates and the samples being tested.

**Table 2:** Settings of experimental testing.

Testing fixture	Stretch value, %	Temperatures, °C	Stretch rate, Hz
Parallel plates setup (diameter = 25 mm and gap thickness = 1 mm)	0.25, 5, 25, and 50	20 and 60	1, 10, and 25

The oscillating shear tests were conducted in a completely reversed strain-controlled mode with a sinusoidal waveform. The asphalt binder specimen was placed between parallel plates (diameter = 25 mm and gap thickness = 1 mm) of the computer-controlled rheometer and subjected to varying strains at different frequencies and temperatures. Stress-strain loops were collected using a computer-controlled MCR 302 rheometer operated in strain-controlled mode. Multiple data points were sampled, representing a single cycle of the corresponding stress paths versus the applied stretch values for each measured real-time stress-strain hysteresis behavior.

## Discussion of experimental stress-strain results and mechanical constitutive modeling

**Fig. 3:**
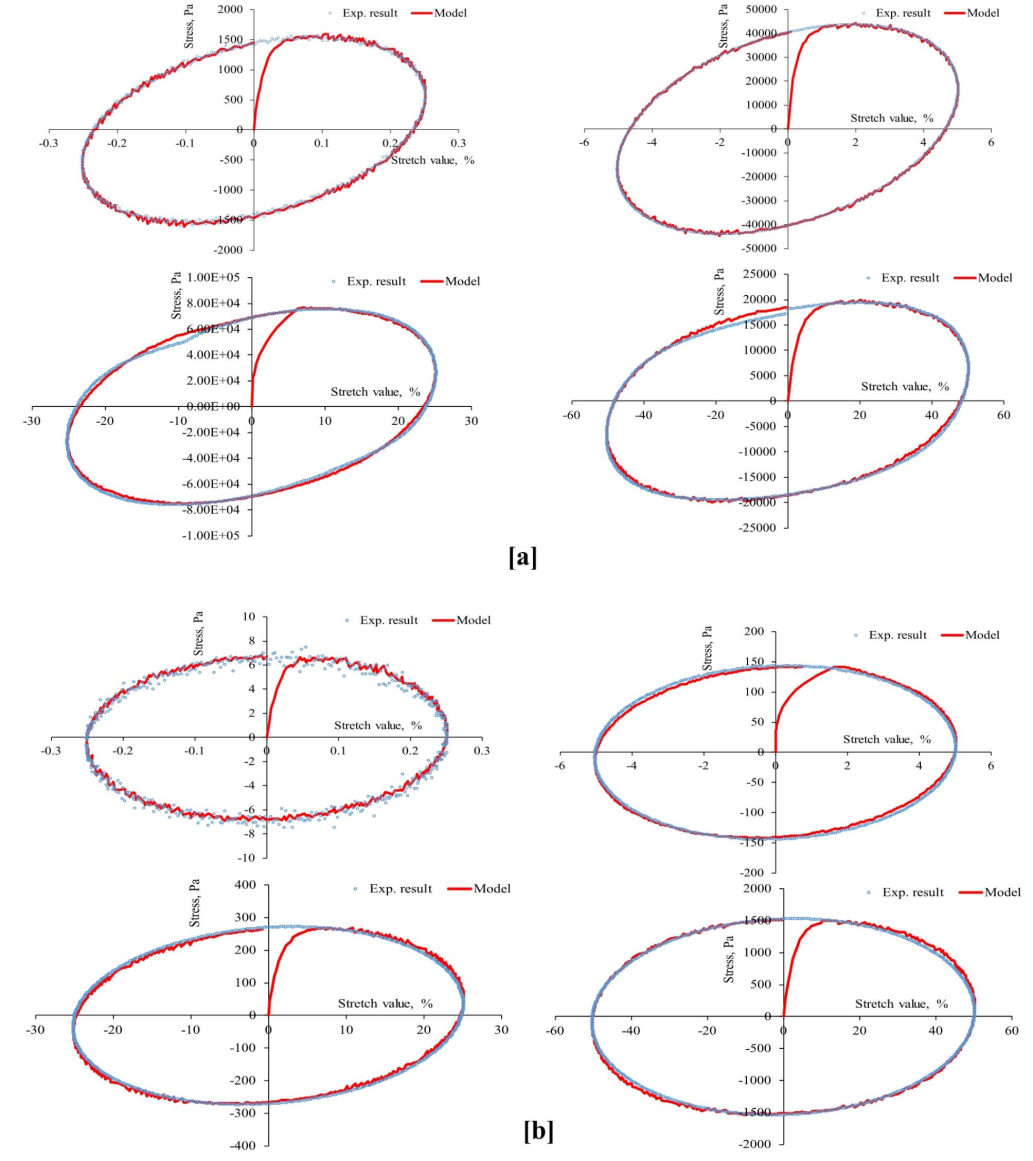
Stress-strain hysteresis loops of unaged binder for 1 Hz stretch rate and stretch values of 0.25, 5, 25, and 50%: (**a**) at 20 °C and (**b**) at 60 °C.

**Fig. 4:**
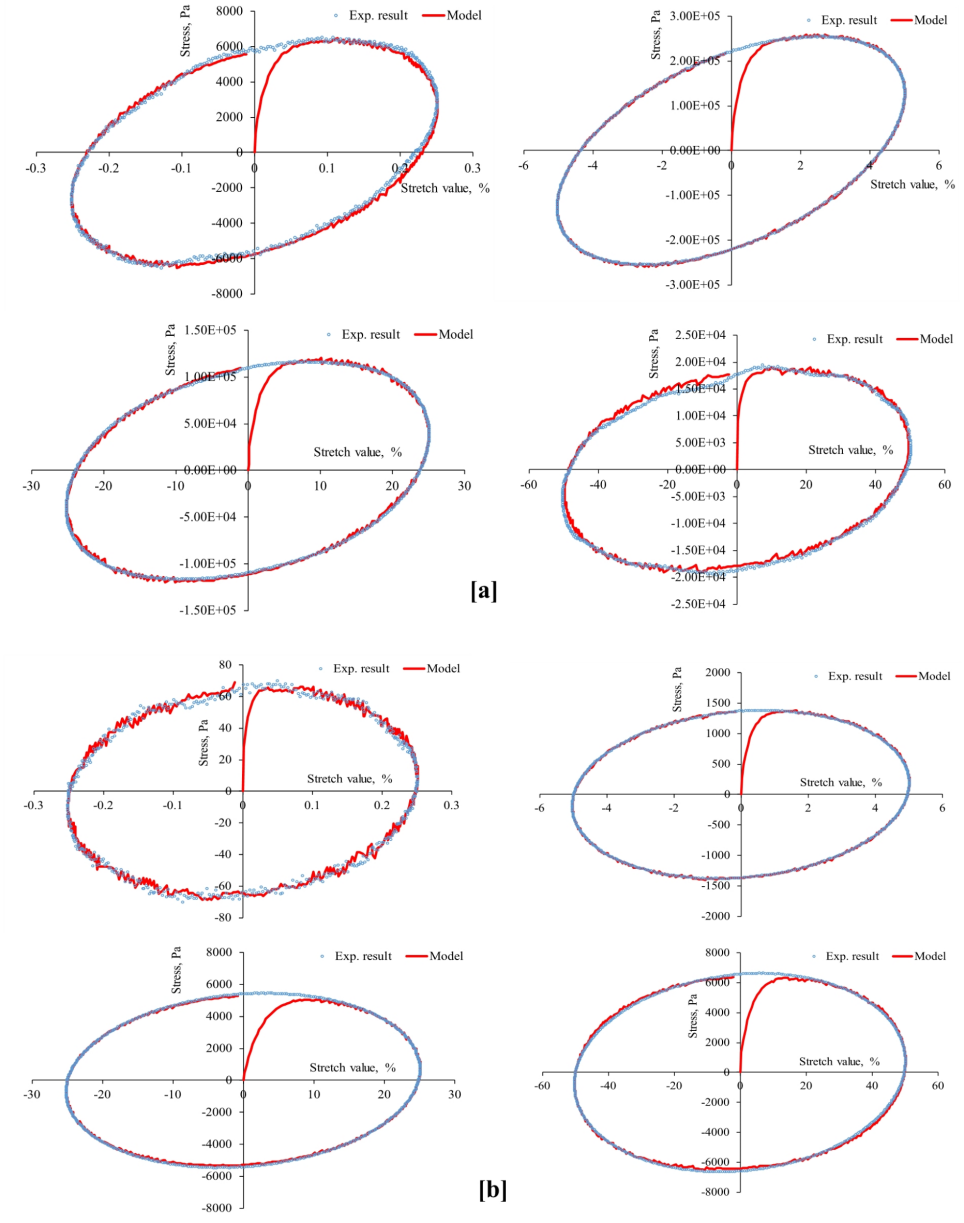
Stress-strain hysteresis loops of unaged binder for 10 Hz stretch rate and stretch values of 0.25, 5, 25, and 50%: (**a**) at 20 °C and (**b**) at 60 °C.

**Fig. 5:**
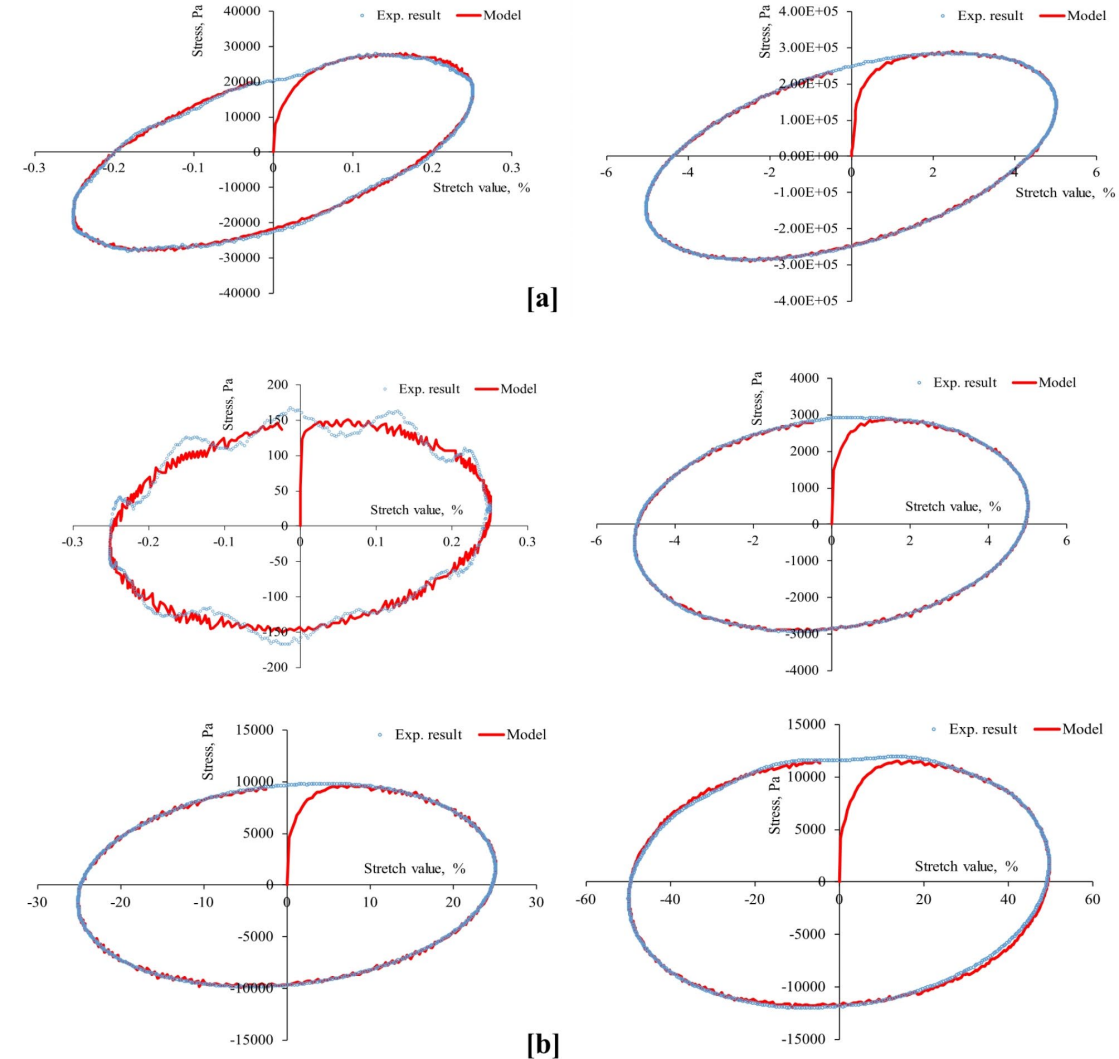
Stress-strain hysteresis loops of unaged binder for 25 Hz stretch rate and stretch values of 0.25, 5, 25, and 50%: (**a**) at 20 °C and (**b**) at 60 °C.

**Fig. 6:**
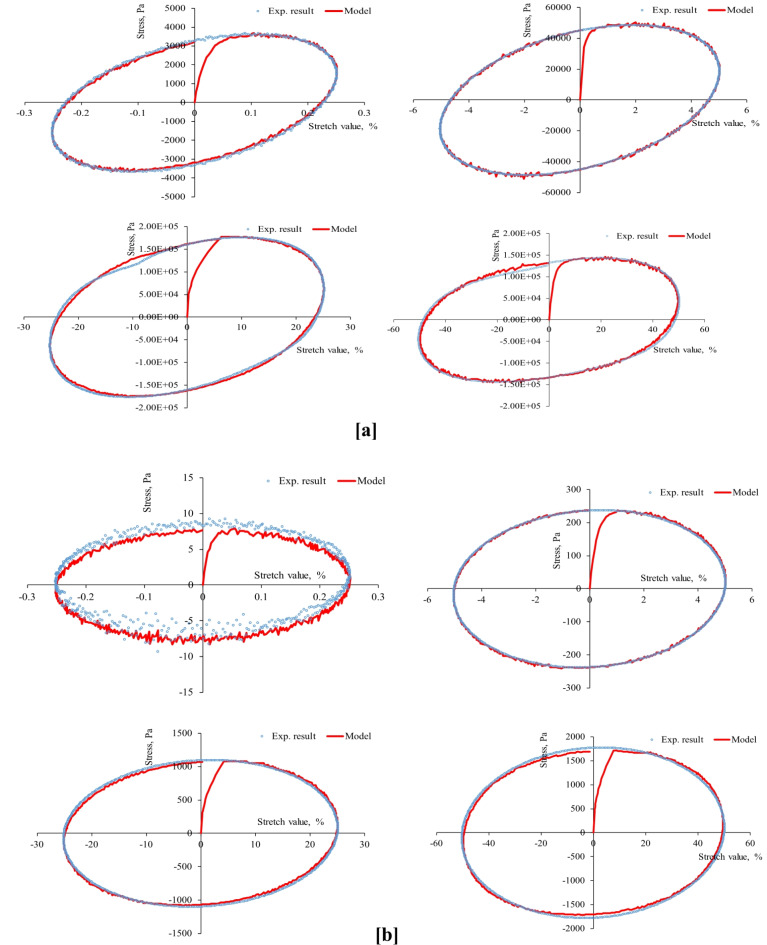
Stress-strain hysteresis loops of RTFO-aged binder for 1 Hz stretch rate and stretch values of 0.25, 5, 25, and 50%: (**a**) at 20 °C and (**b**) at 60 °C.

**Fig. 7:**
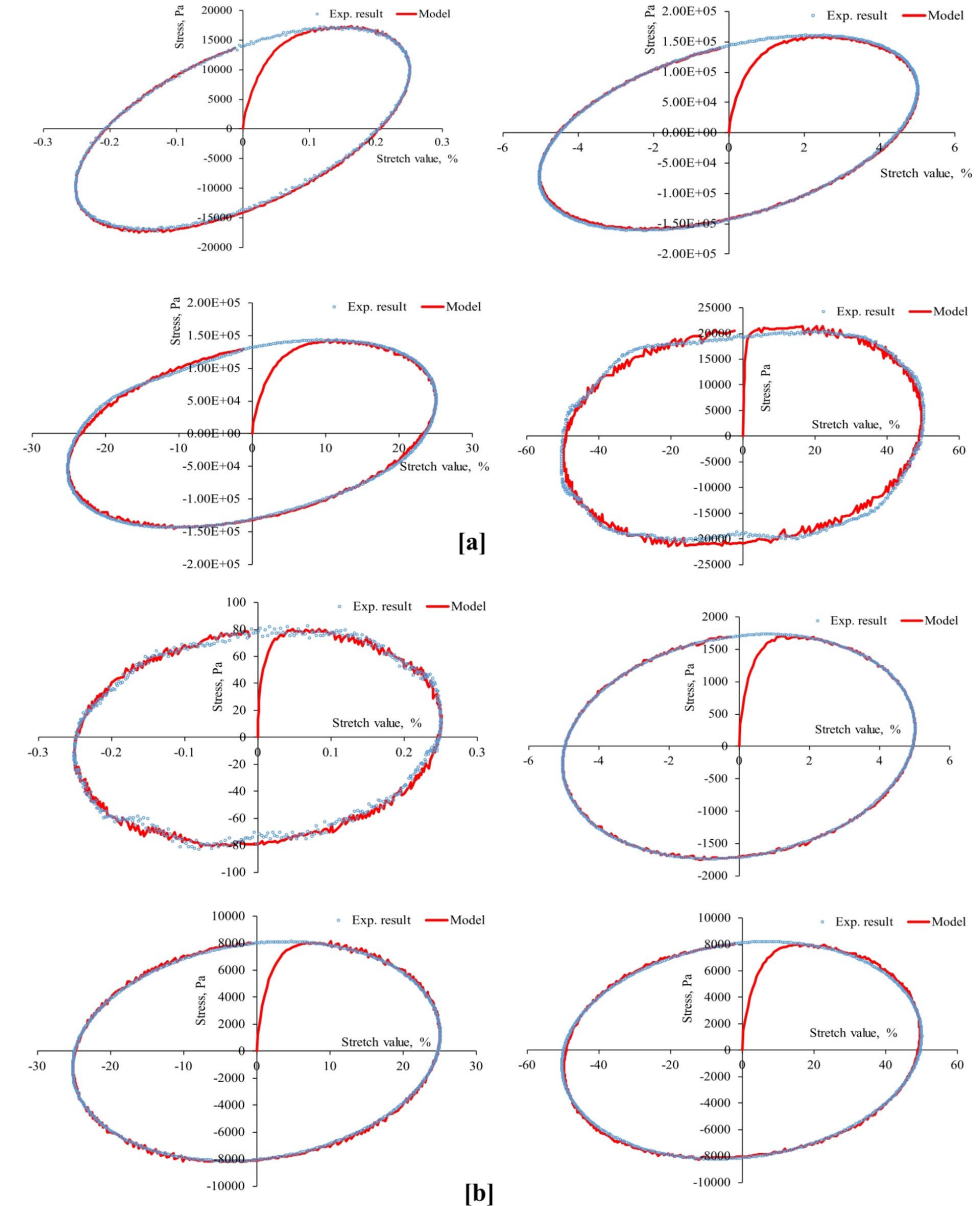
Stress-strain hysteresis loops of RTFO-aged binder for 10 Hz stretch rate and stretch values of 0.25, 5, 25, and 50%: (**a**) at 20 °C and (**b**) at 60 °C.

**Fig. 8:**
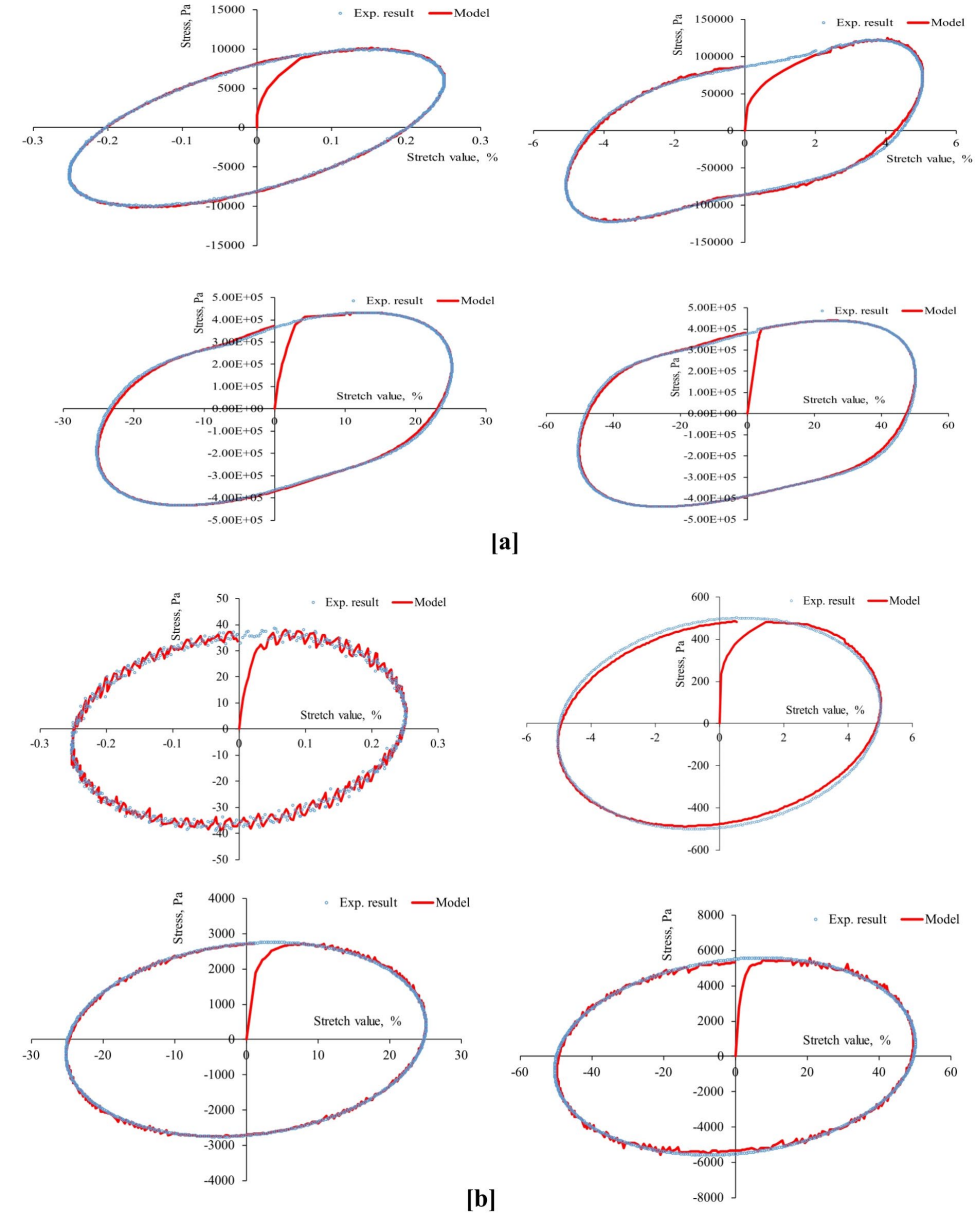
Stress-strain hysteresis loops of PAV-aged binder for 1 Hz stretch rate and stretch values of 0.25, 5, 25, and 50%: (**a**) at 20 °C and (**b**) at 60 °C.

**Fig. 9:**
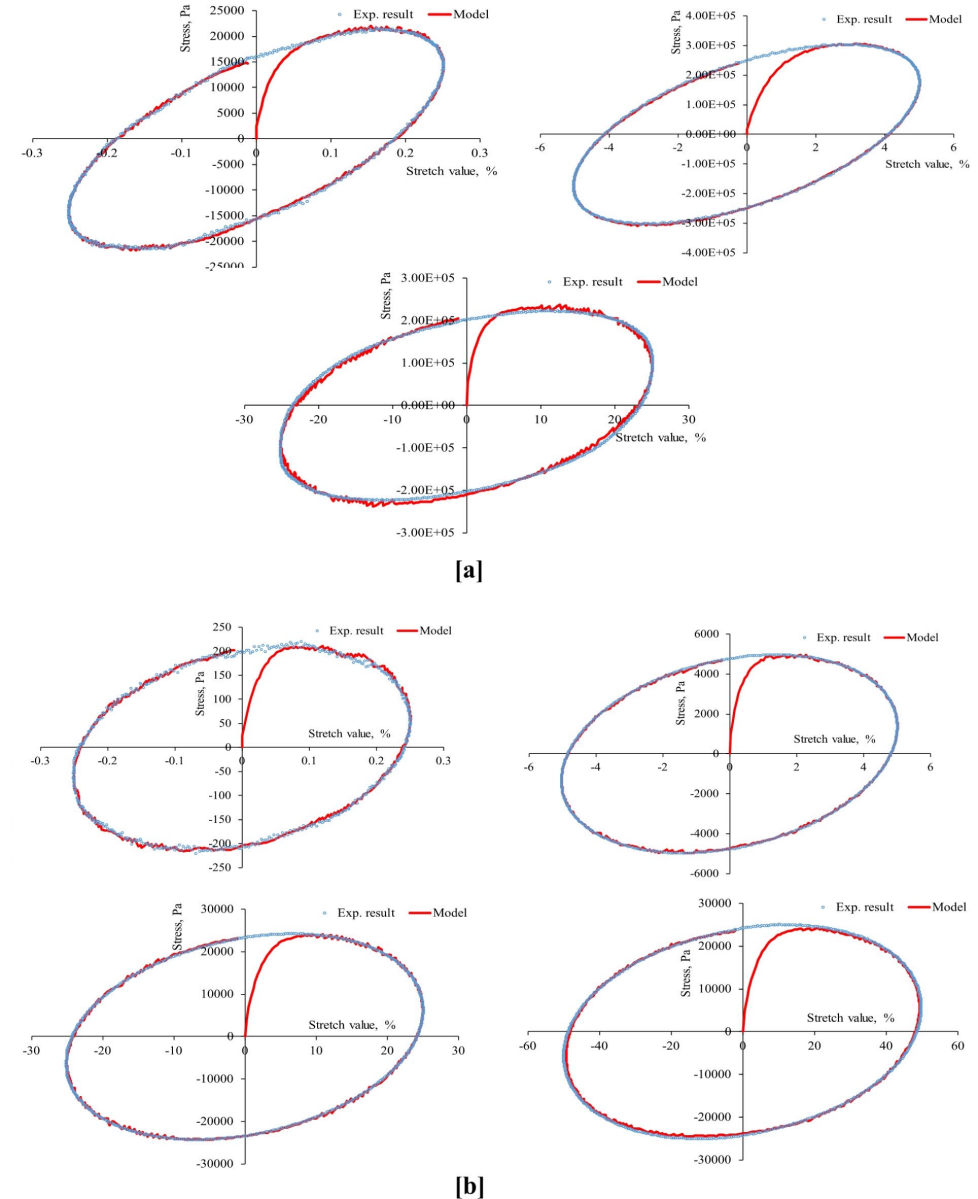
Stress-strain hysteresis loops of PAV-aged binder for 10 Hz stretch rate and stretch values of 0.25, 5, 25, and 50%: (**a**) at 20 °C and (**b**) at 60 °C.

First, the strain-controlled oscillatory shear tests conducted in this study recorded the resulting stresses simultaneously, producing 53 stress-strain hysteresis loops (at three aging levels, four stretch values, two temperatures, and three stretch rates) shown in Figs. 3, 4, 5, 6, 7, 8 and 9. The results highlighted the influence of strain amplitude, temperature, and frequency on the stress-strain orbits of the asphalt binder samples. Noteworthy, the stress-strain loops for the first complete loading cycle demonstrated significant temperature and excitation condition effects on the asphalt materials, exhibiting strain rate dependence within the studied temperatures and deformation ranges.

The stress-strain loops for different excitation conditions revealed that the typical elliptical shape within the linear viscoelastic domain gradually deviated as the strain exceeded the linear viscoelastic strain threshold. At elevated temperatures and ultralow strains (e.g., 0.25%), the rheometer exhibited erratic responses at minimal excitation levels, potentially attributable to the rotor’s insensitivity to such low excitation intensities. The experimentally observed stress-strain responses in this study are consistent with those for different types of binders. Generally, stress decreases as strain level reaches high values in the sample, with each aging state responding differently to oscillating strain, resulting in varied stress-strain loop shapes depending on deformation level, temperature, and loading rate.

At the same excitation conditions, shear stress increased with aging as the material stiffened. Nonlinearity in stress-strain behavior became evident as strain amplitude increased. Experimental investigation indicated different nonlinear stress-strain behavior features in oscillating shear loading. Higher testing frequency or strain rate resulted in greater slopes of the major axis of the stress-strain orbit, indicating more nonlinearity due to greater retarded response. According to AASHTO T 315, the complex shear modulus can drop by a maximum of 5% from its starting value to maintain linearity conditions. Exceeding this strain threshold resulted in nonlinear behavior, visually observed as a non-elliptical stress-strain orbit. Large strain values within the nonlinear regime caused the Lissajous loop to become non-elliptic/non-sinusoidal pattern, with more apparent disfigurement^51^. Worth mentioning, the enclosed area of the stress-strain hysteresis orbit represented energy dissipation under sinusoidal shear motion. Increasing strain resulted in more stiffness loss and nonlinearity in stress-strain. Radical deformity in the loop indicated nonlinear response initiation. This distortion of the stress response and the erratic behavior of the shear stress may arise from several factors: (1) the potential for edge failure, which may reduce the effective sample volume during large strain oscillatory shear measurements, (2) the partial loss of adhesion between the sample and the upper or lower steel plates of the rheometer, (3) internal damage to the sample at elevated strain levels, and (4) the stability of the microstructure throughout each cycle of large strain oscillatory shear measurements may influence the occurrence of stress response distortion.

Second, the proposed parallel rheological framework constitutive model structure was validated using experimental measurements. Measured and predicted stress-strain orbits for one cycle (1st cycle) were graphically compared in Figs. 3, 4, 5, 6, 7, 8 and 9. The modeling results meticulously matched the experimental measurements in both linear and nonlinear conditions. The comparison suggested that the mechanical constitutive model accurately captured the hysteretic behavior of asphalt binders under varying strain rates, amplitudes, and temperatures in oscillatory shear mode. Routine tests within this work scope indicated the practicality of applying this nine-parameter parallel rheological framework model for generalization purposes.

The material property constants of the constitutive model were derived by converging sampling data points from the stress-strain loop with the model under optimal conditions, utilizing an optimization algorithm within the MCalibration software^52^. Initially, a set of parameter values was selected as a starting point for the optimization process. The Levenberg-Marquardt algorithm, among others, was employed to iteratively adjust these parameter values. Once a satisfactory agreement was established between the results of the constitutive model and the experimental data, the model parameters were finalized. The bulk modulus values for both unoxidized and oxidized asphalt binders were determined through molecular dynamics simulations, grounded in the literature, particularly referencing Pan’s study^53^. The model demonstrates versatility, accommodating various bulk modulus values while accurately reproducing the experimental stress-strain hysteresis behavior. The convergence between model predictions and data obtained from DSR tests has validated the model’s efficacy in representing both linear and nonlinear viscoelastic behaviors of asphalt binders. This optimization process yielded a total of 477 values across the nine-parameter model (9 parameters × 53 stress-strain loops).

## Development of the numerical FE model

The numerical modeling aimed to demonstrate the efficacy of extending and integrating the constitutive modeling structure into the finite element (FE) platform to develop a numerical model capable of interpreting the mechanical behavior of asphalt binders under oscillating shear mode. The parallel plate fixture geometry and the sample (25 mm diameter and 1 mm height) were generated in LS-PrePost using single-integration-point hexahedral elements. The specimen consisted of 14,820 elements, each with a height of 0.2 mm.

**Fig. 10:**
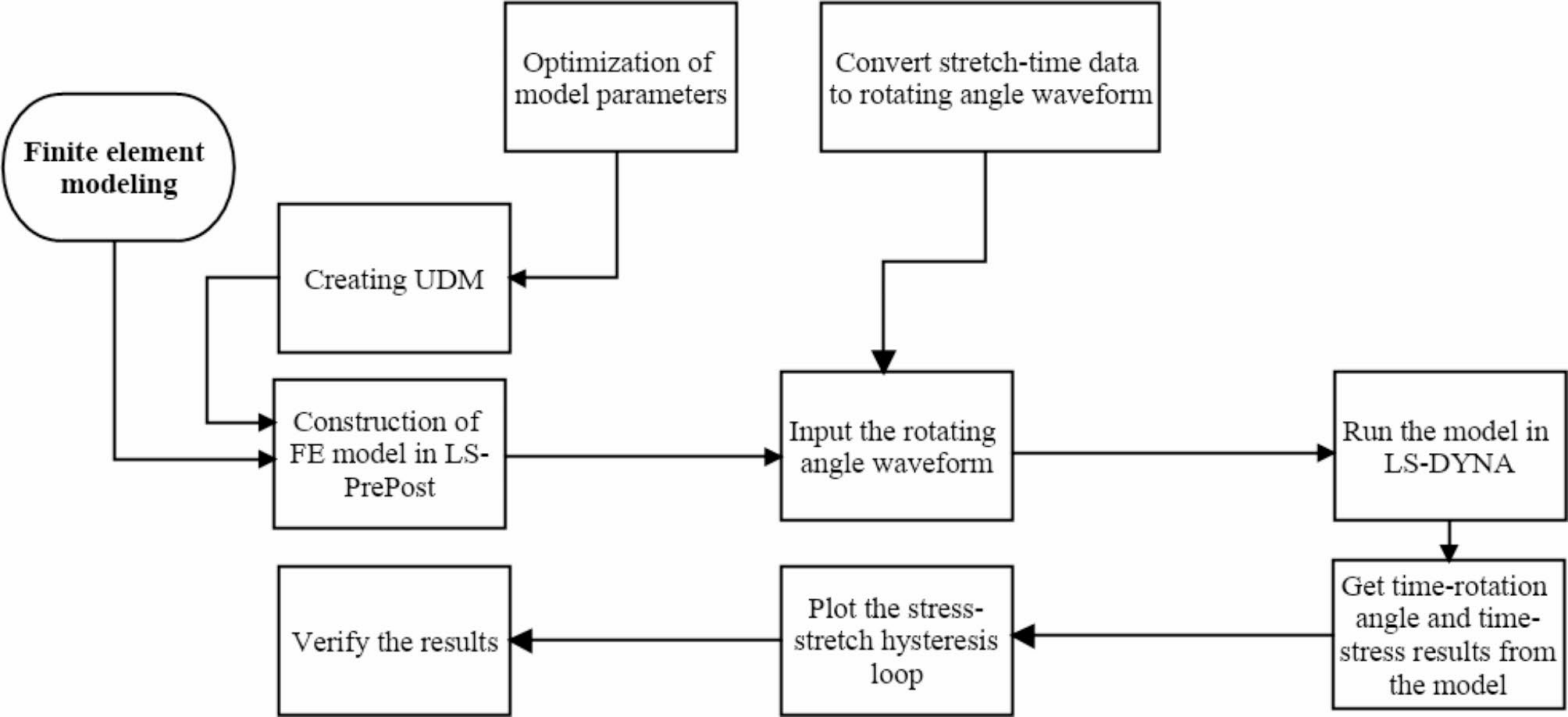
Schematic view of implementing the constitutive model in FE Platform LS-DYNA.

A user-defined material model (UDM) with nine parameters was created and imported into the LS-PrePost model, which was then assigned to the solid elements to invoke the model input parameters and define the material’s behavior for model simulations under stretching conditions. The characteristics of the asphalt binder were incorporated into the UDM parameters. Modifications in each FE model included incorporating the corresponding UDM and imposing rotational shear strain amplitude. The steps of this process are summarized in Fig. 10.

**Fig. 11:**
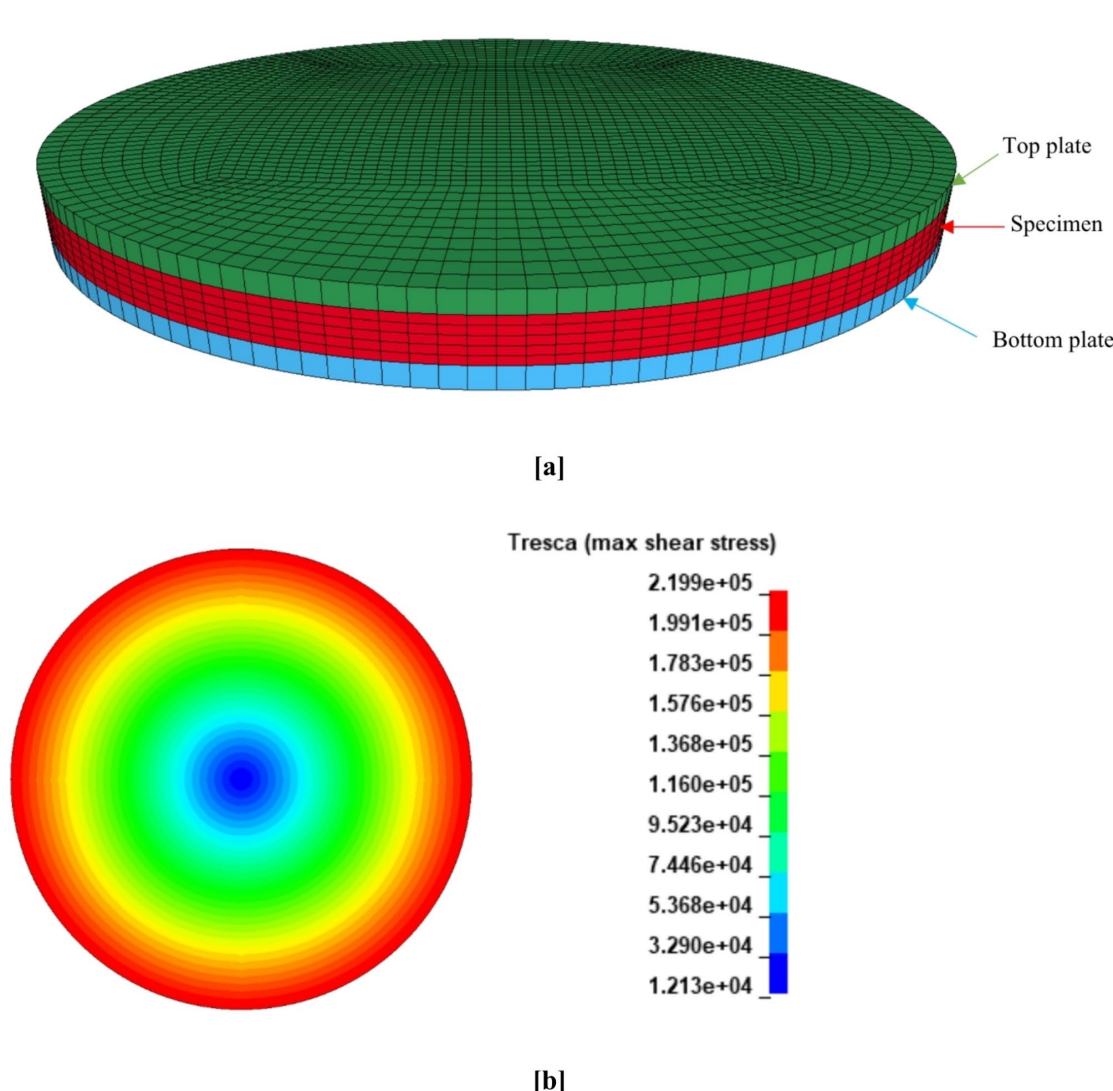
FEM model and shear stress results: (**a**) geometry and meshing of DSR sample, and (**b**) sample stress results (PAV aged residue sample tested at 20 °C, 25% stretch level, and 10 Hz).

The lower and upper plates were modeled as rigid bodies and connected to the specimen at the whole contact interface. The lower plate was clamped, while the top plate was subjected to prescribed sinusoidal strain and frequency. To get the stress values, the torque values imported from the model were then converted to stress using the equation, stress = 2T/(πr^3^), where r is the sample radius and T is the torque. The rotational angle θ obtained from the model at different torque values was converted to strain $\:=r{\theta}/h$, where h is the gap height. All numerical simulations were accomplished using the double precision version of the general-purpose FE solver LS-DYNA R13. Simulations were performed on a local computer with a five-core 2.40 GHz processor and 8 GB RAM. The geometry, meshing, and shear stress distributions for the chosen FE model parameters are presented in Fig. 11.

**Fig. 12:**
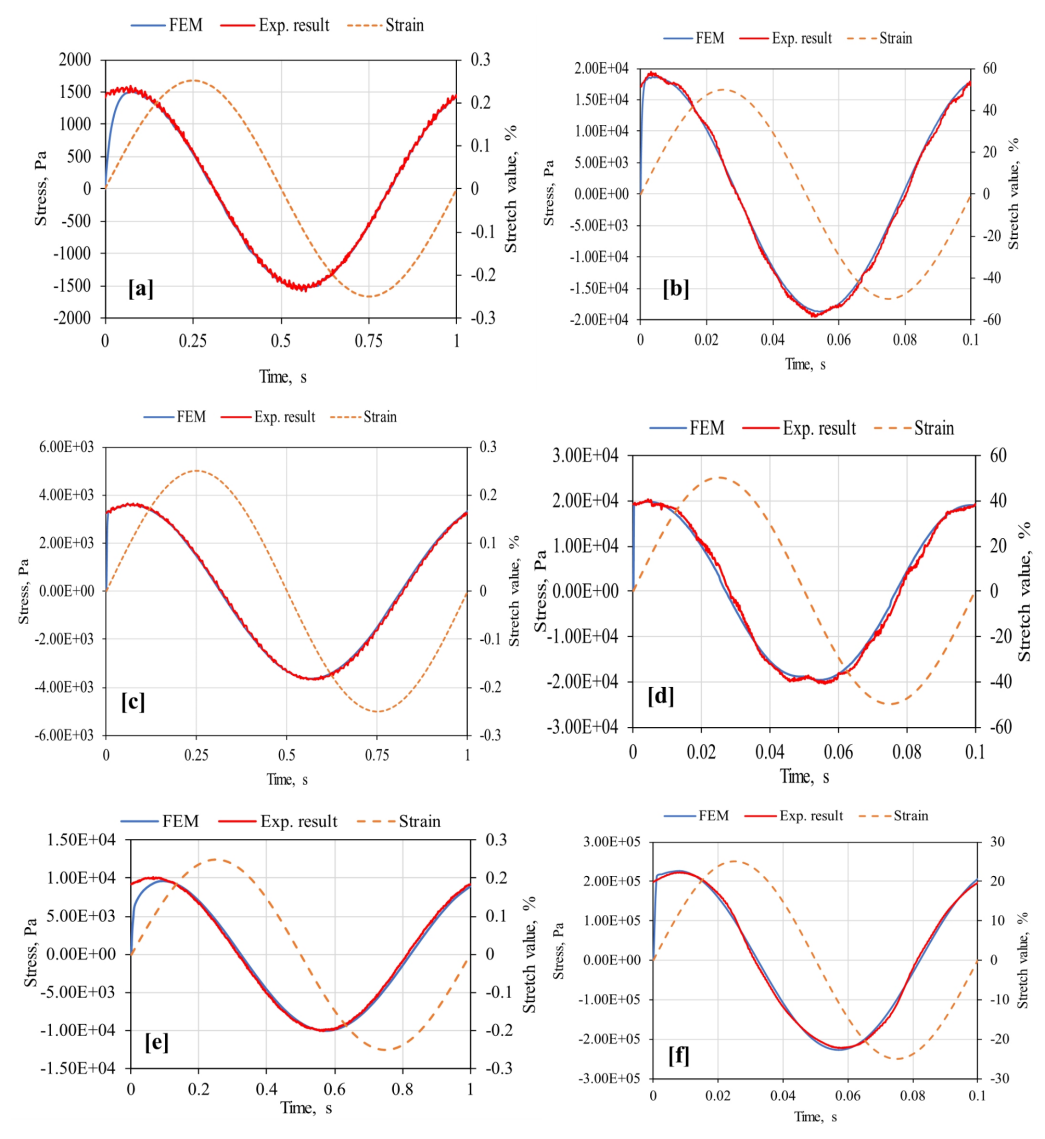
Sample experimental measurements vs. strain and stress waveforms predicted from the numerical simulation of FEM at 20 °C for (**a**,**b**) unoxidized binder, (**c**,**d**) RTFO-aged residue, and (**e**,**f**) PAV-aged residue.

For adequate treatment of the paper length constraint, strain and stress waveforms for randomly selected cases from the 53 model runs were demonstrated in Fig. 12. The accuracy of the FE model predictions was examined using the R-squared values between the predictions and measurements, which reached excellent values, as provided in Table 3. The input (strain) signal was smooth and sinusoidal from start to end of the oscillating cycle. Similarly, the response (stress) sinusoidal waveform was smooth within the linear viscoelastic domain, but soon became distorted, indicating the stress response was no longer proportionally dependent on the exciting strain due to discontinuities from progressing damage in the material. The material’s response to the applied oscillating strain was of the same frequency/time but with a changed phase.

Identifying the onset strain at which the sinusoidal stress transitions to a non-sinusoidal profile was challenging, varying with material characteristics, temperature, and loading rate. Noise in the measurements was evident at ultra-high excitations due to the associated permanent deformation. When the material undergoes permanent deformation, the stress response was not solely dependent on the applied strain, resulting in a more deformed shape.

The numerical modeling of the shear tests yielded encouraging results, indicating the constitutive model had a good capacity for integration and operation in the FE environment, accurately describing the linear and nonlinear viscoelastic behavior of asphalt binders. The numerical simulation demonstrated that the constitutive approach was suitable for incorporation into the FE platform to correctly reproduce the observed stress-strain behavior in shear mode. The proposed FE approach and modeling framework have several potential application directions in asphalt mixtures and pavement structures. For example, the model could be applied to predict the long-term performance of asphalt pavements by accurately simulating the nonlinear viscoelastic behavior of asphalt binders under repeated traffic loads. The framework could be used to develop performance-based criteria for asphalt binders, particularly in terms of stress-strain hysteresis behavior, guiding the selection of binders with enhanced resistance to deformation and aging for specific environmental and loading conditions.

**Table 3:** Comparison of FEM fitting quality to experimental results.

Test conditions				Coefficient of determination, *R*^2^ (FEM predictions vs. experimental measurements)		
Temperature, °C	Stretch level, %		Frequency, Hz	Original samples	RTFO-aged residue	PAV-aged residue
20	0.25		1	0.992	0.980	0.997
			10	0.976	0.982	0.984
			25	0.967	-	-
	5		1	0.996	0.996	0.958
			10	0.983	0.980	0.982
			25	0.967	-	-
	25		1	0.995	0.987	0.973
			10	0.956	0.966	0.958
			25	-	-	-
	50		1	0.993	0.992	0.993
			10	0.980	0.980	-
			25	-	-	-
60	0.25		1	0.979	0.946	0.975
			10	0.971	0.96	0.955
			25	0.945	-	-
	5		1	0.988	0.993	0.983
			10	0.984	0.989	0.968
			25	0.984	-	-
	25		1	0.975	0.963	0.995
			10	0.977	0.966	0.974
			25	0.976	-	-
	50		1	0.989	0.956	0.991
			10	0.981	0.974	0.983
			25	0.966	-	-

“-“ means that the stress-strain hysteresis loop could not be measured due to instability or loss of bonding between steel plates and tested samples.

## Summary and conclusions

This study focused on advancing the understanding and modeling of asphalt binder behavior under oscillating shear conditions, addressing key aspects of both linear and nonlinear mechanical responses. The research contributed significantly by integrating experimental data and numerical simulations to comprehensively characterize asphalt binder rheology, highlighting the following main conclusions:

- The experimental phase captured the complex behavior of asphalt binders across a range of conditions, including strain amplitude, strain rate, temperature, and aging effects. Oscillatory shear tests covered deformation levels from ultra-low to ultra-high strains, generating extensive stress-strain data essential for model development and validation.
- A nine-parameter parallel rheological framework constitutive model was developed, consisting of two networks. Network A used a FEC hyperelastic model to capture the equilibrium response, while Network B combined a FEC hyperelastic model and a linear viscoelastic flow model in series, effectively representing the nonlinear viscoelastic behavior and hysteresis response of asphalt binders.
- Numerical simulations were performed using the LS-DYNA finite element software, integrating the nine-parameter material models into finite element models (FEMs) that mirrored experimental conditions. This computational approach proved both efficient and precise in modeling the complex stress-strain interactions.
- The model was validated with a dataset of 53 FEMs, subjected to various stretch conditions, frequencies, and temperatures, reflecting real-world scenarios. The simulations showed a high correlation with experimental stress-strain responses, confirming the model’s accuracy.
- This study addresses the limitations of existing models focused on linear viscoelasticity, offering a novel framework for accurately modeling both linear and nonlinear viscoelastic oscillatory rheometric stress-strain hysteresis responses of asphalt binders. The findings are significant for improving understanding of binder mechanics and enhancing asphalt pavement durability and performance.

In summary, this study not only expanded the theoretical foundations of asphalt binder rheology but also validated its practical applicability through advanced numerical simulations. The integration of experimental and computational methodologies facilitated a comprehensive exploration of asphalt binder behavior, offering valuable insights for future research and pavement engineering practices.

## Electronic supplementary material

Below is the link to the electronic supplementary material.

Supplementary Material 1

## Data Availability

The datasets used and/or analyzed during the current study are available from the corresponding author on reasonable request.
