# Supplementary material for: Modeling linear and nonlinear viscoelastic oscillatory rheometric stress-strain hysteresis of asphalt binders
Source: Sci Rep. 2024 Nov 18;14:28499. doi: 10.1038/s41598-024-78551-8 (PMC11573977; doi:10.1038/s41598-024-78551-8)
Supplement: Supplementary file 1 — Supplementary Material 1 [file 41598_2024_78551_MOESM1_ESM.docx]

**Highlights**

 Comprehensive analysis of stress-strain hysteresis loops for unaged, short-term aged, and long-term aged asphalt binders across various stretch amplitudes, frequencies, and temperatures.

 Effective capturing both linear and nonlinear viscoelastic behaviors via Nine-Parameter Parallel Rheological Framework.

 Accurate numerical simulations of stress-strain hysteresis responses owing to integrating the developed constitutive model into the LS-DYNA finite element environment.

 Demonstrating high precision in replicating experimentally measured stress-strain loops and validating the proposed model through detailed numerical and experimental comparisons.

 Advancing the understanding of asphalt binder behaviors through providing a robust modeling framework adaptable to various aging states, excitation conditions, and environmental factors.
